# Identification of a new *BRCA2* large genomic deletion associated with high risk male breast cancer

**DOI:** 10.1186/s13053-014-0022-x

**Published:** 2015-01-16

**Authors:** Ana Rafaela de Souza Timoteo, Betina Menezes Albuquerque, Patricia Cristina Pascoto Moura, Carlos Cesar de Oliveira Ramos, Lucymara Fassarela Agnez-Lima, Tom Walsh, Mary-Claire King, Tirzah Braz Petta Lajus

**Affiliations:** Universidade Federal do Rio Grande do Norte, Av. Senador Salgado Filho, s/n, Natal, RN Brazil; Hospital Liga contra o Câncer, Departamento de Mastologia, Natal, RN Brazil; Hospital Liga contra o Câncer, Departamento de Pesquisa Translacional, Natal, RN Brazil; Hospital Liga contra o Câncer, Laboratório de Patologia Cirúrgica, Natal, RN Brazil; University of Washington, Department of Medicine and Genome Sciences, Health Sciences K-160, Seattle, USA

**Keywords:** Male Breast Cancer (MBC), BRCA2 mutation, Next-generation sequencing, Large genomic deletion

## Abstract

**Background:**

Male breast cancer (MBC) is an uncommon disease that has been the focus of limited research. It is estimated that approximately 10% of men with breast cancer have a genetic predisposition, with *BRCA2* being the most prevalent genetic mutation. Here we describe the case of MBC in a 64-year-old man who presented on physical examination a nodule in his left breast and declared to have an extensive family history of cancer.

**Methods and results:**

The patient was firstly diagnosed with an invasive ductal carcinoma (IDC) with histological grade III, nuclear grade 3, pT4N2Mx and positive for hormonal receptors and HER2. Exome sequencing was performed by massive parallel sequencing which had detected a novel *BRCA2* germline mutation that is a large genomic deletion of 3,492 nucleotides including *BRCA2* exon 14, and this deletion is out of frame and is predicted to lead to a stop codon in exon 15 at codon 2,496.

**Conclusion:**

Large rearrangements in *BRCA1* and *BRCA2* occur in a small percentage (<1%) of patients tested for hereditary breast and ovarian cancer. This is the first report of the mutation del3492 in *BRCA2* exon 14, which leads to a truncated protein and therefore is clinically relevant. Mutation segregation analysis should be further done in the Brazilian population. Herein we highlight the importance of next-generation sequencing in the detection of large genomic deletions.

Advances in molecular genetics have made possible to identify a small subset of individual patients and families who have increased cancer risk because of the presence of mutations in cancer susceptibility genes. In the majority of cases however, specific mutations have not yet been identified or cancers have arisen sporadically in the context of various risk factors, including a positive family history [1]. *BRCA1* and *BRCA2* are genes with large coding sequences, consisting of 51 exons in total and a large number of mutations have been characterized worldwide. Together, germline mutations in these two genes are considered to constitute approximately 6–7% of breast cancers and 10% of ovarian cancers, but these numbers vary between populations. More than 2000 different mutations are reported in the Breast Cancer Information Core (BIC) database [2] and the majority of mutations described are frameshift mutations which result in a truncated protein. Male breast cancer (MBC) accounts for less than 1% of all male cancers and 0.65% of all breast tumors [3-5], and men tend to be diagnosed at an older age than women (mean age is about 67 years). It is an infrequent, poorly characterized disease which is epidemiologically and biologically different from female breast cancer (FBC), and it is still unknown whether current paradigms and treatment of female disease can be extrapolated to MBC [6]. Decreased awareness of the existence of such a disease among male patients and physicians leads to its late presentation, where the majority of cases are invasive with distant metastasis and subsequently carry poorer prognoses. The incidence of MBC has increased about 26% over the past 25 years, similarly to FBC [7,8]. Extensive knowledge about female *BRCA1/2* and other inherited germline mutations are available, whereas little is known of male tumors from high-risk families. Besides this, mutation detection and estimation of prevalence of MBC are restricted to North America and Europe, while data from other populations, such as Brazil, are scarce [9]. In a recent article, Thuler and Bergmann have published a retrospective cohort study with clinical-epidemiological characteristics of 1189 Brazilian male breast cancer patients and the results have showed an increase in the incidence and mortality in MBC in Brazil [10] but there is no data concerning molecular aspects. Moreover, the majority of known *BRCA2* mutations are point mutations detected by Sanger sequencing which is unable to detect large deletions and amplifications of complete exons. The present study reports a man harboring a deletion of 3,492 nucleotides in exon 14 of *BRCA2* gene. To our knowledge, this is the first MBC reporting a *BRCA2* mutation in Brazil, showing the absence of this kind of study in this population. This study brings a new insight about methodology used to screen for germline *BRCA2* mutation.

A 64 year-old man reported a painful node in the left breast since 2 years (2008). He related a strong familial breast cancer history (father dead - prostate cancer, mother and six sisters dead - breast cancer, four out of five brothers dead – cancer not identified) (Figure 1). He denied using drugs or anabolic steroids and did not drink large amounts of alcohol. On physical examination, an irregular sub areolar node was detected. Although gynecomastia has been suggested to be present in 6-38% of breast cancer cases in men, it was not evident in our patient [11]. The nipple was not retracted and the left axillary lymphnodes were palpable. The right breast was normal. On sonography a node measuring 3.4 cm with BI-RADS score 4 was detected. After core biopsy, the material was sent for a frozen section (0.6 × 0.5 × 0.6 cm). The diagnosis was invasive ductal carcinoma (IDC) with histological grade III and nuclear grade 3 (Figure 2). The patient did not present himself to the hospital during 1 year. After 1 year, the patient returned to the Medical Service and was submitted to a left radical mastectomy and axillary dissection. Histology confirmed an IDC of 2.5 cm, grade 3 and six out of 23 lymph nodes were positive, staging resulted in pT4N2Mx (tumor, node and metastatic involvement). Tumor size and lymph node involvement are two clear prognostic factors for male patients with breast cancer. Men with tumors measuring 2–5 cm have a 40% higher risk of death than men with tumors <2 cm in maximum diameter. Immunohistochemical analysis showed that the tumor was positive for the presence of estrogen/progesterone receptors and for the human epidermal growth factor receptor-2 (HER-2). The patient initiated adjuvant treatment with 4 cycles of TAC (Taxotere, Adriamycin and Cyclophosphamide). Following adjuvant treatment he was treated with local radiotherapy, dose 5.000 cGy (25 × 200 cGy), followed by a treatment with tamoxifen from 2010 until the present (35 months). The bone scintigraphy showed other pathologic sites at T9, L1, ischium and acetabulum (Figure 3), therefore treatment was associated with pamidronate.

**Figure 1:**
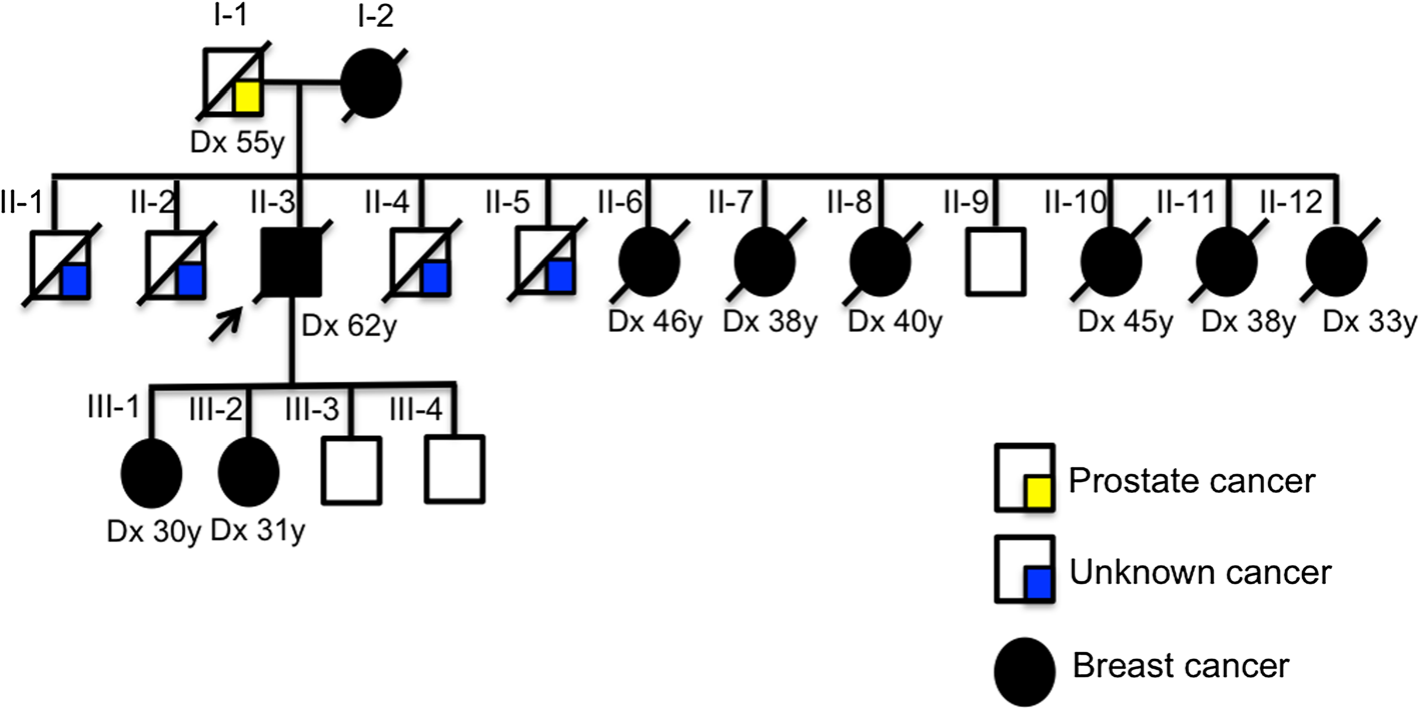
**Patient pedigree.** II.3 is the proband (indicated by an arrow). The figure shows a family history of cancer of 2 generations. Father dead of prostate cancer (I.1). Mother and sisters dead of breast cancer (I.2, II.6, II.7, II.8, II.10, II.11, II.12, respectively) and four brothers dead of unknown cancer (II.1, 2, 4, 5). Two daughters from first marriage also have been diagnosed with breast cancer (III.1 and III.2). The age of diagnostic are indicated (Dx).

**Figure 2:**
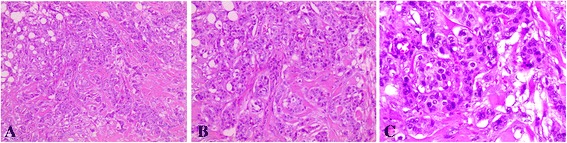
**Hematoxylin and eosin-stained sections of paraffin-embedded tumour biopsy.** The diagnosis was invasive ductal carcinoma (IDC) with histological grade III and nuclear grade 3. Magnifications **A)** 100X **B)** 200x and **C)** 400X.

**Figure 3:**
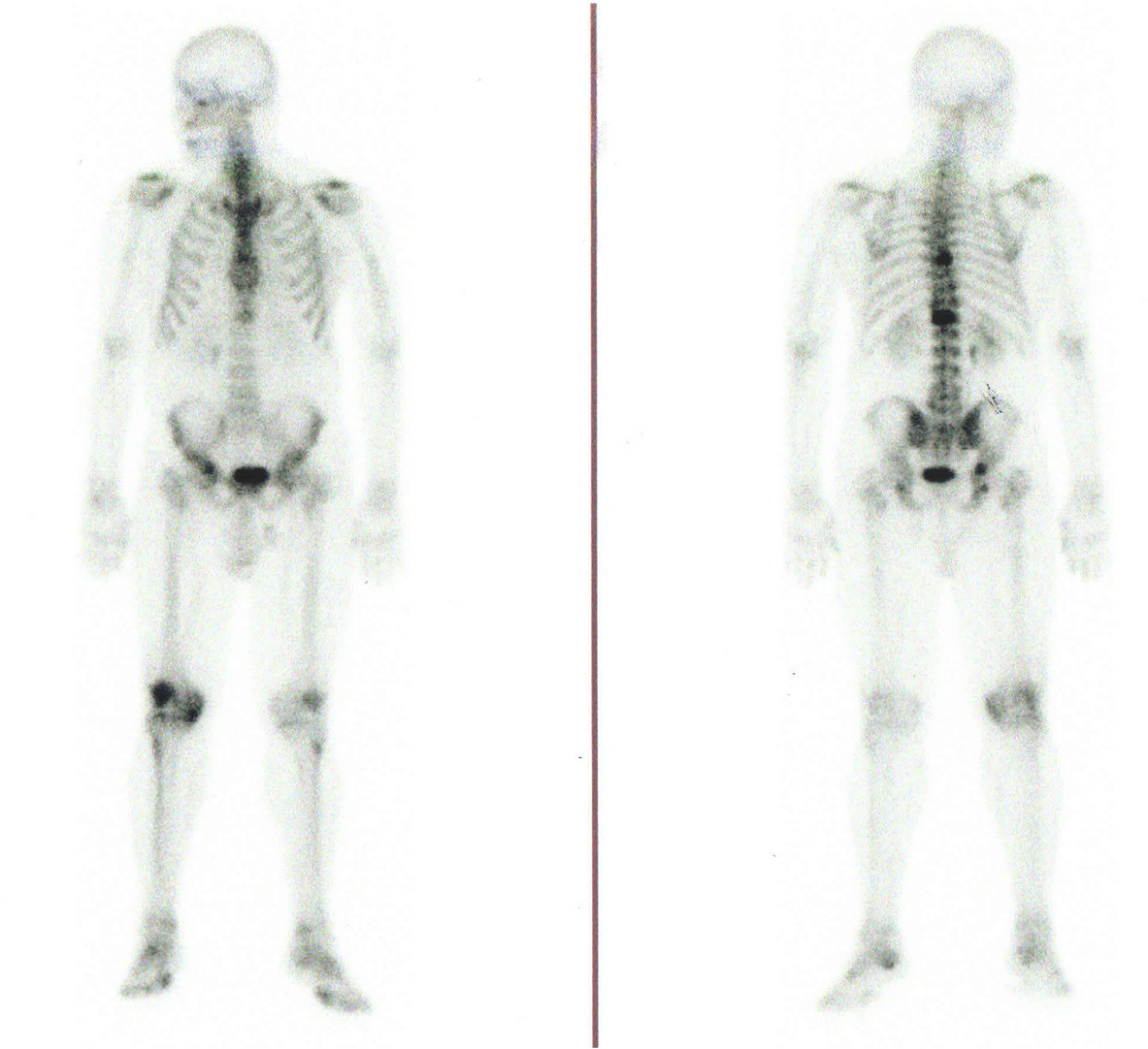
**Proband’s bone scintigraphy realized on December, 2** ^**nd**^ **2009 showing focus of metastasis at T9, L1, ischium and acetabulum.**

Because the patient was an affected individual with MBC and familial breast cancer history, he was referred to a genetic counseling service. Considering his extensive cancer history, a genetic analysis was conducted. DNA was extracted from peripheral blood [12] and exome analysis was performed, applying the BROCA test, which is a targeted capture sequencing approach, to identify all single base pair substitutions, insertion-deletions, and copy number variants in all known breast cancer genes (33 genes) [13,14]. The criteria for genetic screening for breast cancer genetic assessment was conducted based on NCCN guidelines v1.2013 (NCCN Clinical Practice Guideline in Oncology- Genetic/Familial High Risk Familial Assessment: Breast and Ovarian. Version 1.12).

A large heterozygous genomic deletion was identified, predicted by read-depth analysis of BROCA sequence to be chr13: 32,926,826 to 32,930,318 (hg19). A 3,492 nucleotide deletion was detected at *BRCA2* sequence, *BRCA2*_g.26826_30318del, including exon 14, which is out of frame and is predicted to lead to a stop in exon 15 at codon 2,496. To analyze the mutation effect at transcript level, RNA was extracted from FFPE (formalin fixed paraffin embedded) samples of normal and tumour tissue followed by RT-PCR reaction. The PCR was performed utilizing primers against 13 and 15 *BRCA2* exons forward and reverse, respectively (13 F-GCCGATTACCTGTGTACCCT; 15 F-GAAAGACGCGTTGCCTTTGT). No amplification was observed in the proband’s samples while for the controls (70 ng from cDNA from MRC5-V1 cells) a 530 bp product was amplified, as expected (Figure 4), showing that the deletion is present in both tissues. *BRCA2* gene has 27 exons and there are 14,823 mutations related in the BIC database. In *BRCA2* exon 14, there are a total of 624 entries for mutations, of which 76 are distinct mutations, polymorphisms and variants and 40 alterations reported only once. The majority of mutations found to date in the *BRCA1/BRCA2* genes in breast and/or ovarian cancer families are point mutations or small insertions and deletions scattered over the coding sequence and splice junctions. Large rearrangements in *BRCA1* and *BRCA2* occur in a small percentage (<1%) of patients tested for hereditary breast and ovarian cancer [15]. Moreover, the mutation was detected in a male patient.

**Figure 4:**
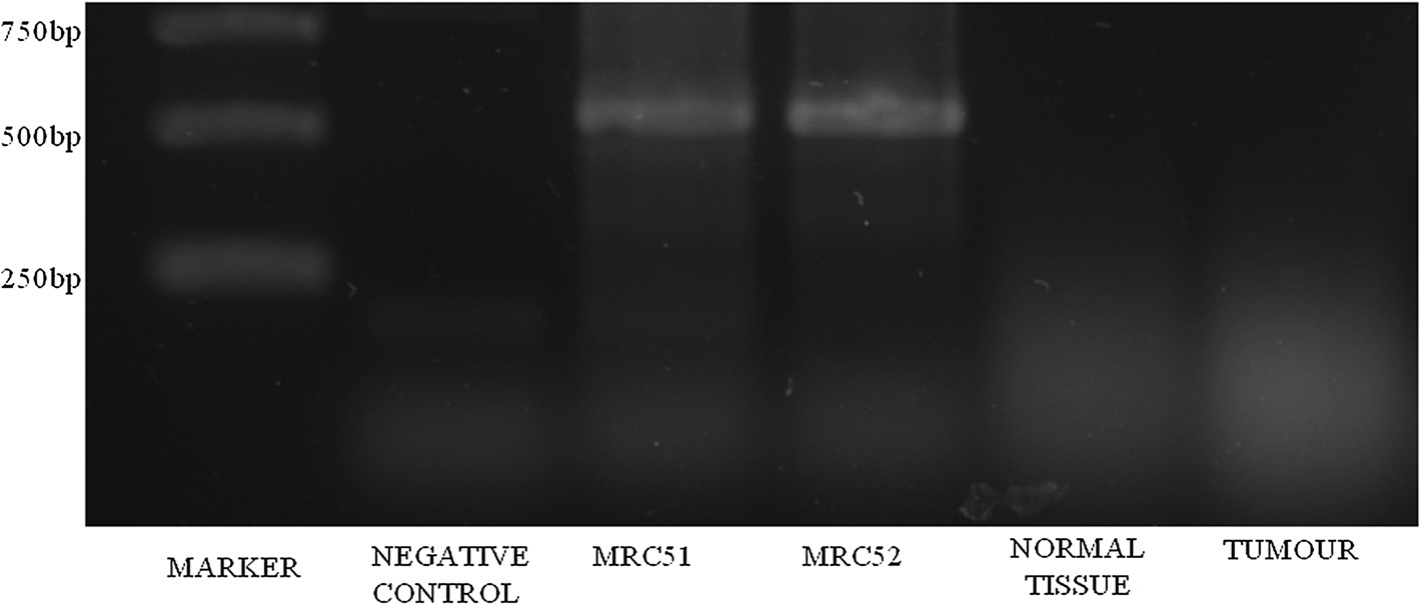
**Mutation effect at transcript level.** Agarose gel (1,5%) showing no amplification of proband’s samples using a forward primer in exon 13 and a reverse primer in exon 15 of *BRCA2.* Total cDNA from two independent RNA extractions from MRC5-V1 cells was used as a positive control for fragment amplification. A total of 70 ng from cDNA was used in each reaction. The length of the fragment expected is 530 bp.

Unfortunately, the patient developed bone metastasis and died of multiple organ failure in July 2013. He was still under treatment with TAC and pamidronate.

The proband had two daughters from a first marriage, whose both had breast cancer, suggesting that this could be a high penetrance mutation, but it was not possible to analyze their DNA. The patient had two sons (Figure 1; III.3 and III.4), both unaffected, the youngest was tested and he does not carry this mutation.

Some authors suggest [16] that testing might be considered in men without cancer, or with the diagnosis of prostate cancer, and a first or second-degree relative diagnosed with breast or ovarian cancer before the age of 50 years. They are taught and encouraged for breast self-examination and to perform twice-yearly clinical breast examinations.

Male breast tumors are usually found by palpation. The most common presentation is a painless subareolar mass (50 – 97%) [17]. About 42% of breast cancer cases in men are diagnosed in advanced stage (III or IV) because men do not seek medical attention for breast masses as quickly as women.

It is estimated that approximately 10% of men with breast cancer have a genetic predisposition, with *BRCA2* being the most prevalent gene mutation and *BRCA1* being less [13,16,18-20]. It is estimated that these mutations contribute to 4 – 40% of hereditary breast cancer in men [21], compared to 5 – 10% of female breast cancer [22]. Furthermore, the life-time risk of breast cancer in male *BRCA2* mutation carriers is approximately 8 – 10 times higher than the general population [23]. *BRCA2* mutations are also associated with increased risks in men for prostate cancer and pancreatic cancer, and possibly increased risks for gallbladder, stomach cancer and melanoma. The occurrence frequency of prostate cancer in males with *BRCA2* mutations is approximately 5 times higher than expected in the general population and the risk to develop pancreatic cancer in males and females with a *BRCA2* mutation is 82.5 times higher and approximately 14 times higher, respectively [24].

Methods to detect germline mutations in high risk families are still too expensive, and in Brazil, the socioeconomic reality is far from this technology. However, next-generation sequencing it is a much more rapid, simple and cost-effective methodology to detect large genomic deletion, instead performing Sanger sequencing and multiplex ligation-dependent probe amplification analysis (MLPA).

The *BRCA2* large heterozygous genomic deletion presented herein is reported for the first time and this can help others to understand the phenotype of individuals carrying this mutation, which is clinically relevant since it leads to a truncated protein. Finally, this result will contribute to create awareness for physicians to investigate for germline mutation in men with a family history of cancer and increase the information of the type of mutation that can be related to breast cancer development in men.

## Consent

Written informed consent was obtained from the patient for publication of this Short report and any accompanying images. A copy of the written consent is available for review by the Editor of this journal.

## Abbreviations

MBC: Male breast cancer
FBC: Female breast cancer
NGS: Next-generation sequencing
BIC: Breast cancer information core
